# Development of a primary care-based complex care management intervention for chronically ill patients at high risk for hospitalization: a study protocol

**DOI:** 10.1186/1748-5908-5-70

**Published:** 2010-09-21

**Authors:** Tobias Freund, Michel Wensing, Cornelia Mahler, Jochen Gensichen, Antje Erler, Martin Beyer, Ferdinand M Gerlach, Joachim Szecsenyi, Frank Peters-Klimm

**Affiliations:** 1 Department of General Practice and Health Services Research, University Hospital Heidelberg, Voßstrasse 2, 69115 Heidelberg, Germany; 2 Scientific Institute for Quality of Healthcare, Radboud University Nijmegen Medical Centre, P.O. Box 9101, 6500HB Nijmegen, Netherlands; 3 Institute of General Practice, Friedrich Schiller University Jena, Bachstraße 18, 07743 Jena, Germany; 4 Institute of General Practice, Theodor-Stern-Kai 7, 60590 Frankfurt am Main, Germany

## Abstract

**Background:**

Complex care management is seen as an approach to face the challenges of an ageing society with increasing numbers of patients with complex care needs. The Medical Research Council in the United Kingdom has proposed a framework for the development and evaluation of complex interventions that will be used to develop and evaluate a primary care-based complex care management program for chronically ill patients at high risk for future hospitalization in Germany.

**Methods and design:**

We present a multi-method procedure to develop a complex care management program to implement interventions aimed at reducing potentially avoidable hospitalizations for primary care patients with type 2 diabetes mellitus, chronic obstructive pulmonary disease, or chronic heart failure and a high likelihood of hospitalization. The procedure will start with reflection about underlying precipitating factors of hospitalizations and how they may be targeted by the planned intervention (pre-clinical phase). An intervention model will then be developed (phase I) based on theory, literature, and exploratory studies (phase II). Exploratory studies are planned that entail the recruitment of 200 patients from 10 general practices. Eligible patients will be identified using two ways of 'case finding': software based predictive modelling and physicians' proposal of patients based on clinical experience. The resulting subpopulations will be compared regarding healthcare utilization, care needs and resources using insurance claims data, a patient survey, and chart review. Qualitative studies with healthcare professionals and patients will be undertaken to identify potential barriers and enablers for optimal performance of the complex care management program.

**Discussion:**

This multi-method procedure will support the development of a primary care-based care management program enabling the implementation of interventions that will potentially reduce avoidable hospitalizations.

## Background

Healthcare systems are faced with an increasing number of patients with complex care needs, resulting from multiple co-occurring medical and non-medical conditions [1,2]. Co-occurrence of multiple chronic conditions is known to influence both clinical practice patterns and health outcomes [3]. Individuals with multiple chronic conditions are more likely to be at risk for functional impairment [4] and adverse drug events [5]. Their medical care is often fragmented by poor coordination between different healthcare providers [3]. Self management capabilities decline with an increasing number of co-occurring medical conditions [6]. Therefore, it is not surprising that patients with multiple chronic conditions are more likely to be hospitalized for a potentially 'avoidable' cause (*e.g*., unmanaged exacerbation, intermittent infection or falls, imperfect transitional care), leading to suboptimal health outcomes and substantial healthcare costs likewise [7].

**Figure 1:**
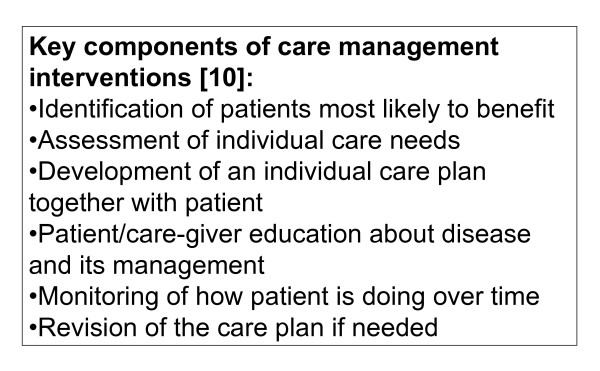
**Key components of care management interventions**. Key components of care management interventions as proposed by Bodenheimer and Berry-Millet [10].

Primary care offers the opportunity to deliver efficient, continuous, and coordinated chronic care. Different authors have made suggestions how primary care can enhance the organization and delivery of chronic illness care [8,9]. In most proposals, care management programs are seen as a promising approach to improve quality of care and reduce costs [10]. These programs are designed to assist patients and their support systems in managing medical and non-medical conditions by individualized care planning and monitoring (Figure 1). Patients with a predicted high risk of future healthcare utilization, but manageable disease burden, were found to benefit most from these programs [10,11].

Therefore, it is crucial to identify as precisely as possible patients most likely to benefit from these programs. Finding high-risk patients in computerized medical record systems, using predictive modelling, has been evaluated in care management trials in the USA and is seen to have better results than case finding by doctors or patient surveys [12,13]. These software models rely on clinically- and cost-similar disease categories called diagnostic cost groups (DCG) [14] or adjusted clinical groups (ACG) [15] that are generated from insurance claims data.

In Germany, chronic heart failure (CHF), chronic obstructive pulmonary disease (COPD), and type 2 diabetes mellitus (DM) were among the 20 most frequent causes for hospital admission in 2008 [16]. All three conditions are stated as being 'ambulatory care sensitive conditions' (ACSC), meaning that primary care has a dominating role in preventing hospital admissions for these conditions [17]. Hospitalisations may be avoidable by coordinated and structured chronic care. Many of the high-risk patients suffering from any of these index conditions will have additional co-morbidities [18,19]. Complex care management may meet disease-specific as well as generic care needs resulting from such co-morbidity. Our goal is to develop a complex care management intervention for patients with any of these conditions (CHF, COPD or DM) and an (estimated) high risk for hospitalization in order to implement intervention elements (*e.g*., self management support, structured follow-up) that may reduce the number of (avoidable) hospitalizations.

As a first step, we plan to adapt complex care management to the specific characteristics of primary care in Germany. Chronic care in Germany is mainly delivered by small primary care practices: The practice team usually consists of one or two physicians (general practitioner or general internist) and a small number of healthcare assistants (HCAs), who have few clinical tasks. HCAs are trained in a three-year part-time curriculum in practice and vocational school. Despite some recent approaches to involve HCAs in chronic care [20], their work is focused on clerical work (including reception) and routine tasks like blood sampling or recording electrocardiograms. However, recent trials on primary care-based disease-specific care management interventions involving trained HCAs show promising results [21-23]. Moreover, practice teams experience the expanded role of healthcare assistants as valuable improvement of chronic care [24-26]. Whereas international research on care management has mainly focused on nurse-led programs, evidence about the potential role of HCAs in chronic care is scarce.

Our overall aim of reducing avoidable hospitalizations by introducing a HCA-led care management intervention targeting patients at high risk for future hospitalization is challenging. Therefore, we plan to study the mechanisms of avoidable hospitalizations due to index and co-occurring conditions. We have to understand how professional and patient behaviour as well as care organization contributes to avoidable hospitalizations and to what extent care management may be able to implement strategies that target the revealed mechanisms. As implementation of an innovation generally faces various problems [27], it is crucial that barriers to change are addressed [28].

The aim of this paper is to describe the study protocol for the development of a complex HCA-led care management intervention for chronically ill patients that aims to implement strategies to reduce avoidable hospitalizations in German primary care.

## Methods

The development uses a framework that is proposed by the Medical Research Council (MRC) for the design and evaluation of complex interventions [29,30]. Based on theories (phase 0/I) as well as our own experience and exploratory studies (phase II) for causes of and solutions for the problem of avoidable hospitalizations, we plan to build an explanatory model of how the planned care management intervention could help to implement strategies to reduce them. It is planned that the model would then be tested and refined. The two phases will be elaborated below.

### Theory and modelling

Phase 0/I involves planning and evaluating complex improvement strategies for patient care and benefits from careful and comprehensive theoretical framing [31,32]. Its main objective is to identify factors that enable or inhibit improvement in patient care.

To develop an explanatory model for the planned care intervention, we will perform a comprehensive literature review on research about avoidable hospitalizations in primary care as a starting point aimed to answer the following questions: What are causes and predictors of avoidable hospitalizations in primary care patients with DM, COPD, and CHF? And which pathways are already known to make care management interventions effective in avoiding these hospitalizations?

To answer question one, we will begin with an expert panel including generalists and specialists on causes of hospitalizations for the index conditions. As a result of the expert panel, we expect to be able to refine our search strategies for the following systematic literature search in Medline. It can be assumed that we will identify some generic causes of hospitalizations for all index conditions. Therefore, we aim to perform in-depth literature searches for identified disease-specific as well as generic causes of hospitalisations. For all literature searches, Medline will be searched via Pubmed. Searches will not be restricted by language, study type, or publication date. Reference lists of retrieved articles will be searched in order to avoid missing relevant evidence. The screening of abstracts and full texts will be performed by one researcher. We aim to end up with a narrative review on existing evidence to answer our research questions.

The effects of primary care-based care management interventions for chronic diseases (question two) will be determined as a result of a comprehensive systematic review and meta-analysis. The details of this review have been published elsewhere [33].

After concluding existing evidence we will consider appropriate theories [31] that may help to explain and predict the effects of the care management intervention on avoidable hospitalizations. It can be assumed that the intervention will have to implement strategies on three levels of care: the behaviour of care providers (*i.e*., general practitioners, specialists, and HCAs), patients, and the organization of healthcare.

For now, the Chronic Care Model (CCM) acts as a first framework for practice redesign in order to enhance quality of care [8]. The components of the planned care management intervention can be structured with the core domains of the CCM (see Table 1).

**Table 1:** Elements of the planned care management intervention

Chronic Care Model Element	Planned care management component
Clinical information systems	Software-based case finding (predictive modelling) Recall-reminder in electronic medical records
Self management support	Collaborative goal setting and action planning, individualized care plans Patient education (symptom monitoring checklist, advise how to deal with deterioration of symptoms)
Decision support	Provider training (GP) on guidelines for the treatment of index conditions/adjustment of treatment regimens in case of co-occuring conditions Provider training on polypharmacotherapy in the elderly
Community resources	Link to existing local resources (*e.g*., smoking cessation programs, physical exercise programs, self-help groups)
Delivery system design	Involvement of HCAs in assessment and proactive telephone follow up Collaborative discharge planning between hospital doctors and GPs/HCAs
Healthcare organization	Financial incentives for HCAs and GPs

### Exploratory studies

As a second step, we plan to perform Phase II exploratory studies to refine our modelled care management intervention with a focus on its implementation in German primary care by answering the following research questions: How can we identify patients most likely to benefit from the planned care management intervention? How can the identified patient population be described regarding healthcare needs and resources? And what are potential barriers or enablers for the implementation of the care model in primary care practices?

### Sampling of practices

We will recruit 10 general practices in Baden-Württemberg (Germany) that care for patients insured by the Allgemeine Ortskrankenkasse (AOK), the general regional health fund. All participating general practitioners (GP) have to be enrolled in the AOK GP-centred healthcare contract [34], which implies that they are the gate-keeping primary care provider for contracted beneficiaries. Other inclusion criteria are: one full-time working GP (or general internist) and at least one full-time working healthcare assistant. We aim to invite all contracted GPs of the region of Northern Baden, Germany. The practice sample will be stratified between single-handed and group practices and will include practices serving rural as well as urban areas.

### Sampling of patients

As case finding is crucial for effective care management we will take two different approaches to invite patients for the exploratory studies:

1. Predictive modelling: We will assess the likelihood of hospitalization (LOH) for all patients from participating practices based on insurance claims data including hospital and ambulatory diagnosis. The software package 'Case Smart Suite Germany' (CSSG 0.6, DxCG, Munich, Germany) will be used for this purpose. CSSG prediction software is based on diagnostic cost groups, demographic variables, and pharmacy data. It has previously been adapted for AOK beneficiaries. Patients with a LOH score above the 90th percentile (LOH^high^) will be invited to participate in the study if at least one of the index conditions (COPD, CHF, or DM type 2) is present. In order to evaluate the impact of depression as co-occurring condition, patients with minor or major depression aged 60 years and older will also be included in the exploratory studies if predicted as LOH^high ^patients (by CSSG). Minors (age <18 years), patients living in nursing homes or receiving palliative care will be excluded from the study. Dialysis and current treatment for cancer (defined as ongoing chemotherapy or radiotherapy) account for extreme high LOH scores and are therefore added as exclusion criteria.

2. GP selection: In addition to the first approach, GPs will be asked to propose eligible patients themselves. They will be instructed to choose only patients who are rated as being at high risk for future hospitalization and are seen as being likely to benefit from a care management intervention (same inclusion and exclusion criteria as mentioned above). GPs will be blinded about the LOH score until their proposal has been submitted to the study centre.

These studies will serve as a pilot for recruitment for the future trial on care management. The three identified patient populations (software selection only, GP selection only, selected by both) will be compared regarding morbidity burden and treatment patterns (analysis of claims data) as well as healthcare needs and resources (patient survey and chart review). This comparison may help us to develop an optimal approach to identify susceptible patients with high risk for future healthcare utilization, but still manageable for primary healthcare teams.

Patients from both subpopulations will be invited by their treating GPs and will have to give written informed consent prior to final inclusion in the study. It is planned to recruit a total number of 200 participating patients.

### Insurance claims data analysis

It can be assumed that most of the identified patients will suffer from more than the index condition. Insurance claims data will therefore be analysed to assess co-morbidity and its patterns in LOH^high ^patients. Co-occuring medical conditions will be assessed by condition count, Charlson comorbidity score [35], and cluster analysis. We will further assess hospital admissions and costs for patient subgroups based on morbidity and LOH score. Because adverse drug events resulting from polypharmacy are known to be one potential cause of avoidable hospitalizations [5], we plan to assess treatment pattern in LOH^high ^patients using pharmacy data. They will be compared to guideline recommendations with regard to co-occurring medical conditions. We will use descriptive statistical methods (*e.g*., frequencies, cross-tables) to evaluate and interpret insurance claims data.

### Patient survey

LOH^high ^patients and patients proposed by the GP will be invited to participate in the patient survey. It consists of a paper-based questionnaire with different measures for patients' medical and non-medical needs and resources (Table 2). We aim to assess patients' resources and perceptions of patient-provider interactions (medication adherence, beliefs about medication, salutogenic and social resources, health locus of control) as well as care needs (alcohol abuse, depression) in order to inform tailoring of the model of care. We will use descriptive statistical methods and regression models for the detection of independent associations (if appropriate) in order to detect additional intervention targets.

**Table 2:** Content of patient questionnaire

Dimension	Measuring instrument
Socio-demographic data	Single items from a German standard questionnaire [37]
Perceived burden of disease	self-developed questionnaire
Quality of Life	EuroQol (EQ-5D) [38]
Depression	PHQ9 [39]
Adherence	MARS [40]
Beliefs about medication	BMQ [41]
Sense of coherence	SOC [42]
Health locus of control	KKG [43]
Social support	FSozU K22 [44]
Substance abuse	CAGE [45]
Healthcare climate	HCCQ [46]

### Chart review and physician survey

GPs will document computer-based case report forms (CRFs) for every participating patient. The CRF contains physician ratings regarding patients' morbidity, needs and resources, and treatment (Table 3). Throughout this survey, we will be able to assess the validity of diagnostic codes from insurance claims data by comparing them to physician-rated morbidity. Furthermore, we gain detailed clinical data on the severity of index and co-occurring conditions. Because patient-provider concordance may impact on quality of care for LOH^high ^patients, we aim to compare physicians' and patients' ratings of existing conditions, medication adherence, social support, and health behaviour.

**Table 3:** Content of physician questionnaire

Dimension	Measuring instrument
Comorbidity	CIRS [47]
Rating of patients' adherence	self-developed instrument
Rating of patients' self-care and health behavior	self-developed instrument
Rating of patients' social support	self-developed instrument
HbA1c, creatinine [Diabetes Patients]	patient chart
FEV1 [COPD Patients]	patient chart
Ejection fraction [CHF Patients]	patient chart
Current Medication	patient chart

The remote data entry system uses Pretty Good Privacy (PGP)-encrypted SSL technology for secure transmission of the data from the questionnaire.

### Qualitative studies

#### Interviews with GPs

We will use in-depth interviews with GPs to explore and discuss causes of avoidable hospitalizations of participating patients, and how they could have been prevented by implementing a new care model. Therefore, we plan to review distinct hospital admissions due to ambulatory care sensitive conditions (ACSCs) identified by the analysis of insurance claims data of patients from the GP's list. Barriers and enablers for implementation will additionally be explored throughout the interviews by describing the care management process in detail.

#### Focus groups with healthcare assistants

All HCAs from participating practices will be invited to a focus group discussion about the feasibility of the planned care management intervention. Barriers and enablers for future implementation will be explored by discussing a detailed description of the planned care management intervention (*i.e*., paper case with care management process).

#### Interviews with patients

Participating patients from the survey will be asked to take part in a semi-structured interview about their medical and non-medical care needs. We will further explore how they experience hospitalizations and what they would expect from and fear of a care management intervention.

All topic guides for the three qualitative studies will be developed by a multi-disciplinary board of health services researchers and include GPs, nurses, and sociologists. All interviews and focus groups will be performed by skilled interviewers or moderators and digitally audio-taped. The material will be transcribed verbatim and analysed using qualitative content analysis [36].

#### Ethics

The studies comply with the Helsinki Declaration 2008. Ethical approval was granted by the ethical committee of the University Hospital Heidelberg (S-052/2009) prior to the beginning of the studies.

## Discussion

HCA-led primary care-based interventions that target chronically ill patients at high risk for future hospitalisation are an interesting and challenging new approach. We have described the steps that inform the development and design of such a care model: Prior to the evaluation regarding effectiveness, we aim to explore underlying mechanisms of avoidable hospitalizations and how they may be targeted. Additionally, qualitative studies with practice teams and patients will inform about barriers and enablers of the implementation of the care intervention. We aim to end up with a detailed model about how the planned care management intervention may work, and how its components may feasibly be implemented in daily practice.

## Competing interests

The project is funded by the general regional health funds (AOK). All authors declare that funding will not influence the interpretation and publication of any findings. Michel Wensing is an Associate Editor of Implementation Science. All decisions on this manuscript were made by another Senior Editor.

## Authors' contributions

TF is responsible for the design of the study and wrote the first draft of the manuscript. FPK, CM, AE, MB, FMG, JG, and SZ participated in the design of the study and revised the manuscript critically. All authors read and approved the final manuscript.
